# Severity distribution and treatment of chronic obstructive pulmonary disease in China: baseline results of an observational study

**DOI:** 10.1186/s12931-022-02021-w

**Published:** 2022-04-29

**Authors:** Ting Yang, Baiqiang Cai, Bin Cao, Jian Kang, Fuqiang Wen, Yahong Chen, Wenhua Jian, Hongyan Shang, Chen Wang

**Affiliations:** 1 Department of Pulmonary and Critical Care Medicine, China-Japan Friendship Hospital, Beijing, 100029 China; 2 National Clinical Research Center for Respiratory Diseases, Beijing, 100029 China; 3 Department of Respiratory and Critical Care Medicine, Peking Union Medical College Hospital, Beijing, China; 4 Department of Respiratory and Critical Care Medicine, The First Hospital of China Medical University, Shenyang, China; 5 Department of Respiratory and Critical Care Medicine, West China Hospital, Sichuan University, Chengdu, China; 6 Department of Respiratory and Critical Care Medicine, Peking University Third Hospital, Beijing, China; 7 State Key Laboratory of Respiratory Disease, Guangzhou Institute of Respiratory Disease, National Clinical Research Center for Respiratory Disease, 1st Affiliated Hospital of Guangzhou Medical University, Guangzhou, China; 8 Department of Medical Affairs, AstraZeneca China, Shanghai, China

**Keywords:** Chronic obstructive pulmonary disease (COPD), Disease burden, COPD severity, Maintenance therapy, COPD management, Observational study, China, Outpatients

## Abstract

**Background:**

Chronic obstructive pulmonary disease (COPD) receives low awareness and is undertreated in China. Understanding the burden and treatment of COPD across the nation is important for improving quality of care for this disease. This study aims to reveal the current situation of COPD severity distribution and management across China.

**Methods:**

Baseline data from REALizing and Improving Management of Stable COPD in China, a multicentre, prospective, longitudinal, observational study, were analysed. Patients diagnosed with COPD as per Global Initiative for Chronic Obstructive Lung Disease 2016 (GOLD 2016) criteria were enrolled from 50 randomly selected hospitals (tertiary, 25; secondary, 25) across six geographical regions. Data were collected in routine clinical settings.

**Results:**

Between 15 December 2017 and 6 August 2020, 5013 patients were enrolled and 4978 included in the full analysis set. Of these, 2459 (49.4%) reported ≥ 1 exacerbation within 12 months prior to study enrolment, with a mean annual rate of 0.9/patient, including 0.2/patient and 0.5/patient leading to emergency room visits and hospitalisation, respectively. Spirometry graded 458 (10.1%), 1886 (41.7%), 1558 (34.5%), and 616 (13.6%) were GOLD stage I–IV, and 536 (11.4%), 1034 (22.0%), 563 (12.0%), and 2566 (54.6%) were classified as GOLD 2016 Group A–D, respectively, without evident regional variations. Inhaled corticosteroids plus long-acting beta_2_-agonist (ICS/LABA, 1316 [26.4%]), ICS/LABA plus long-acting muscarinic antagonist (ICS/LABA + LAMA, 871 [17.5%]), and LAMA (754 [15.1%]) were prescribed at high rates across all groups and regions. Medications not recommended by GOLD were commonly prescribed (TCM, 578 [11.6%]; others, 951 [19.1%]), and 681 (13.7%) were not given ICS or long-acting bronchodilators.

**Conclusions:**

Disease burden among Chinese COPD outpatients is high. Improved guideline adherence for COPD treatment is needed.

*Trial registration* ClinicalTrials.gov identifier, NCT03131362.

**Supplementary Information:**

The online version contains supplementary material available at 10.1186/s12931-022-02021-w.

## Background

Chronic obstructive pulmonary disease (COPD) is a progressive respiratory disease that represents a disproportionally high health burden in China compared with around the world. In China, it has a higher prevalence (8.2–13.7% based on population surveys in China [1–3] vs a global age-standardized prevalence of 3.2% [male]/2.0% [female] based on systematic reviews [4]) and higher mortality rate (age-standardized death rate for COPD, 79.4 per 100,000 in China vs 50.7 per 100,000 globally in 2013 [5]). In 2016, the estimated global prevalence of COPD was 251 million cases; with a population of 1397 million in China, the estimated prevalence would suggest between 113 and 187 million of the global cases being in China [1–3, 6, 7]. A number of 910,809 deaths due to COPD occurred in China in 2013, which accounted for about one-third of COPD-related deaths in the world [5]. Even worse, it is projected to affect more people in the next decade due to the ageing population and increasing exposure to risk factors [8].

COPD is manifested by a wide spectrum of symptoms, varying by individual and changing over time [9]. Whilst stable symptoms persist, acute episodes of exacerbations occur intermittently, reducing patient’s quality of life and even leading to morbidity and mortality. The Global Initiative for Chronic Obstructive Lung Disease (GOLD) spirometric grading system classifies airflow limitation into four stages. For a thorough assessment of disease severity and risk of exacerbations, the combined COPD assessment system endorsed by GOLD 2017 Report is a more comprehensive tool [9, 10]. It integrates symptoms, airflow obstruction, and risk of exacerbations and offers a credible framework for stratifying patient and guiding treatment decisions. The Chinese Thoracic Society are responsible for national guidelines for COPD management and recommend the GOLD diagnosis and management strategy document [11].

Nationwide surveys revealed a diagnosis rate as low as 1% among Chinese patients with COPD [1]. Of those diagnosed, approximately 11.7% received medications, including medications not intended for COPD treatment [1]. Nonadherence to GOLD strategy document has been noted by previous studies, including the indiscriminate use of inhaled corticosteroids (ICS) [12], frequent use of short-acting bronchodilators [13], and prescription of medications beyond the recommended list [14, 15].

China has a three-tier healthcare system, where specialist care is provided by secondary and tertiary hospitals but qualified personnel and advanced facilities are concentrated in the tertiary hospitals. Poor guideline awareness, variations in institutional clinical practice, and high cost of some recommended medications in some areas such as those in the rural regions may hamper widespread adherence to the GOLD strategy document in China. The regional disparity in healthcare quality and lack of proper diagnosis and treatment of COPD may be associated with the high mortality rate of COPD in China [5]. The first step to changing the status quo is a comprehensive knowledge of real-world clinical practice for managing COPD around China, which would provide a basis for policy making and resource allocation.

Here we present the baseline results from a multicentre, prospective, observational study on a nationally representative sample of Chinese COPD outpatients, providing insights into the characteristics, severity distribution, and treatment situation of COPD in China.

## Methods

### Study design and patients

This reflects the methodology to obtain the baseline data from the REALizing and Improving Management of Stable COPD in China (REAL; ClinicalTrials.gov: NCT03131362). This is a multicentre, prospective, observational study that aims to understand the distribution, clinical course and management of COPD in China. Hospitals were selected from six geographical regions using a multistage, stratified, and cluster sampling approach. Subject enrolment, assessment, and data collection took place in routine clinical settings, without additional intervention to participants.

Subjects were consecutively screened and enrolled during routine clinical visits to outpatient departments. Key inclusion criteria were: outpatients; (1) aged ≥ 40 years; (2) diagnosed with COPD as per GOLD 2016 criteria, based on a post-bronchodilator fixed ratio of forced expiratory volume in 1 s (FEV_1_)/ forced vital capacity (FVC) < 70%; (3) symptoms characteristic of COPD; and (4) a history of exposure to risk factors [9]. In order to include patients with stable COPD at enrolment, those experiencing acute exacerbations within 4 weeks of enrolment were excluded. A target sample size of 5000 subjects was determined, based on the primary objective of this study.

Detailed information on the sampling method, eligibility criteria, and sample size calculation has been reported [16].

### Ethics approval and consent to participate

Written informed consent was obtained from all participants. The Ethics Committee of the China-Japan Friendship Hospital, the leading site, approved the study protocol and informed consent form (ICF) prior to study initiation (approval number 2016-97). All patients participating in this study voluntarily signed the ICF and received a copy before study initiation. The study was approved by ethics committees at individual study centres and performed in full conformance with the Declaration of Helsinki and Good Clinical Practice.

## Outcomes

The primary objective of this longitudinal study was to observe the 1-year clinical outcomes of COPD under routine clinical treatment. Objectives addressed in this baseline study were to: (1) gain a cross-sectional, nationwide view of COPD severity distribution, by GOLD stages and GOLD groups (pre-planned analysis as per GOLD 2016 criteria [9] and post hoc as per GOLD 2017 criteria [17] as defined in GOLD 2017 Report [10]); (2) reveal the pharmacological maintenance therapies used for routine treatment of COPD in clinical practice, across China, focussing on the prescriptions drug class for the GOLD groups (primarily by GOLD 2016 groups and exploratorily by GOLD 2017 groups) and by geographical region; and (3) describe the main non-pharmacological management approaches for COPD.

## Data source and assessment

Baseline data were collected during patient’s first study-related outpatient visit, scheduled by investigators for routine clinical care. Baseline data collection was completed in January of 2019. Nonclinical and clinical data were collected from medical records, physician evaluations, and recorded in a case report form (see published methodology [16]). COPD severity, including airflow limitation severity and combined COPD assessment, were evaluated by investigators according to GOLD 2016 criteria [9]. Symptomatic assessment was based on both the COPD Assessment Test (CAT) and the modified Medical Research Council (mMRC) scores (more symptoms were defined by either CAT ≥ 10 or mMRC ≥ 2). Post hoc analysis was performed on COPD assessments according to GOLD 2017 criteria [17].

### Statistical analysis

Analysis was performed on the full analysis set (FAS), including all participants fulfilling eligible criteria and successfully enrolled. Descriptive statistics were applied to the baseline data. No hypothesis testing was performed. Kappa coefficient was calculated to assess the agreement between CAT and mMRC as a symptom rater to classify GOLD groups. Baseline data was analysed by urban and rural regions and secondary and tertiary hospital setting. Statistical analysis was performed with Statistical Analysis System (SAS) statistical software, version 9.2 or later.

## Results

### Patient characteristics

Between 15 December 2017 and 6 August 2020, 5097 patients were screened at 50 hospitals (25 tertiary and 25 secondary hospitals) across six geographical regions of China. Of these, 5013 were enrolled and 4978 (2597 and 2381 from tertiary and secondary hospitals, respectively) included in the FAS. Baseline post-bronchodilator spirometry measurements were available for 4903 (98.8%) patients.

Most patients were male (79.5%), with a mean age of 66.2 years (SD 8.9; Table 1). Patients were almost equally sampled from urban and rural areas (55.0% vs 45.0%). They had relatively low levels of education, with 1800 (36.2%) having completed primary and 1665 (33.5%) completed secondary school. Most patients’ family incomes fell within a low-to-middle range (< 3021 USD/year, 1260 [25.3%]; 3021‒22 659 USD/year, 3376 [67.8%]). Patients with a smoking history constituted a large proportion (previous, 2556 [51.3%]; current, 1142 [22.9%]).

**Table 1:** Patient characteristics

Baseline characteristics	N = 4978
Male, n/N (%)	3959/4978 (79.5)
Age (years), mean (SD)	66.2 (8.9)
BMI (kg/m^2^), mean (SD)	23.0 (3.6)
Region of residence, n/N (%)	
North	1005/4978 (20.2)
Northeast	623/4978 (12.5)
East	1248/4978 (25.1)
South central	904/4978 (18.2)
Southwest	596/4978 (12.0)
Northwest	602/4978 (12.1)
Residence area, n/N (%)	
Urban	2735/4972 (55.0)
Rural	2237/4972 (45.0)
Highest education, n/N (%)	
Illiterate	165/4976 (3.3)
Primary school	1800/4976 (36.2)
Secondary school	1665/4976 (33.5)
High/technical school	779/4976 (15.7)
University/college	556/4976 (11.2)
Graduate and above	11/4976 (0.2)
Annual per-capita household income (USD),^a^ n/N (%)	
< 3021	1260/4978 (25.3)
3021–22 659	3376/4978 (67.8)
22 659–45 317	278/4978 (5.6)
45 317–75 528	35/4978 (0.7)
> 75 528	29/4978 (0.6)
Occupation, n/N (%)	
White collar worker	379/4978 (7.6)
Blue collar worker	1685/4978 (33.8)
Government officer	139/4978 (2.8)
Unemployed	1233/4978 (24.8)
Retired	2047/4978 (41.1)
Smoking status, n/N (%)	
Non-smoker	1280/4978 (25.7)
Current smoker	1142/4978 (22.9)
Former smoker	2556/4978 (51.3)
Passive smoker	1409/4975 (28.3)
Pack-years,^b^ mean (SD)	42.1 (24.1)
Exposure to noxious particles or gases, n/N (%)	
No exposure	3397/4978 (68.2)
Dust	996/4978 (20.0)
Harmful gas	404/4978 (8.1)
Biofuels	564/4978 (11.3)
Other noxious substances	73/4978 (1.5)
A family history of respiratory disease, ^c^ n/N (%)	1715/4978 (34.5)

All percentages were calculated based on patients with available data

*SD* standard deviation, *BMI* body mass index

^a^Income was queried on a RMB basis and grouped into five intervals (< 20,000 RMB, 20,000–150,000 RMB, 150,000–300,000 RMB, 300,000–500,000 RMB, and > 500,000 RMB), which were herein converted to USD using the 2018 yearly average exchange rate 6.62 and rounded up

^b^Pack-years data were missing for 16 patients

^c^Reported respiratory disease history of patient’s first-degree relatives

Almost half the patients experienced at least one exacerbation during the previous 12 months (2459 [49.4%]; Table 2), at an average rate of 0.9 (SD 1.5) per patient per year. Exacerbations leading to emergency room visits and hospitalisation occurred at an average rate of 0.2 (SD 0.6) and 0.5 (SD 0.9) per patient per year, respectively. Blood eosinophil counts for 564 patients showed that 378 (67.0%) had ≥ 100 cells/μL and 109 (19.3%) ≥ 300 cells/μL.

**Table 2:** Clinical characteristics of patients at baseline

Clinical characteristics	N = 4978
Time since COPD diagnosis (years),^a^ mean (SD)	3.8 (6.2)
Diagnosed as chronic bronchitis, n/N (%)	3295/4978 (66.2)
Diagnosed as emphysema, n/N (%)	3204/4978 (64.4)
COPD signs and symptoms, n/N (%)	
Shortness of breath	2864/4975 (57.6)
Wheezing	3296/4975 (66.3)
Chest tightness	3324/4975 (66.8)
Cough	4037/4975 (81.1)
Mucus purulence	4011/4975 (80.6)
Blood eosinophil counts ≥ 100 cells/μL, n/N (%)	378/564 (67.0%)
Blood eosinophil counts ≥ 300 cells/μL, n/N (%)	109/564 (19.3%)
CAT score,^b^ mean (SD)	14.6 (7.6)
mMRC score,^b^ mean (SD)	1.4 (1.0)
COPD-Q score,^c^ mean (SD)	5.9 (2.0)
Patients with exacerbation in previous 12 months, n/N (%)	2459/4978 (49.4)
Annual exacerbations per patient, mean (SD), range	0.9 (1.5), 0–20
Annual exacerbations leading to outpatient visit per patient, mean (SD), range	0.3 (1.0), 0–11
Annual exacerbations leading to emergency room visit per patient, mean (SD), range	0.2 (0.6), 0–14
Annual exacerbations leading to hospitalisation per patient, mean (SD), range	0.5 (0.9), 0–10
Concurrent respiratory diseases,^d^ n/N (%)	1288/4978 (25.9)
Asthma	437/4978 (8.8)
Respiratory infection	422/4978 (8.5)
Bronchiectasis	151/4978 (3.0)
Non-respiratory comorbidities,^d^ n/N (%)	1981/4978 (39.8)
Hypertension	1028/4978 (20.7)
Coronary artery disease	313/4978 (6.3)
Diabetes mellitus	247/4978 (5.0)
Benign prostatic hyperplasia	168/4978 (3.4)
Chronic gastritis	111/4978 (2.2)

All percentages were calculated based on patients with available data

*CAT* COPD Assessment Test, *COPD* chronic obstructive pulmonary disease, *mMRC* modified Medical Research Council, *COPD-Q* COPD knowledge questionnaire, *SD* standard deviation

^a^Time since COPD diagnosis was missing for 26 patients

^b^CAT and mMRC data were missing for two patients

^c^COPD-Q scores were missing for five patients

^d^Diseases with a prevalence > 2%

Patients on average scored 5.9 (out of 13) on the COPD knowledge questionnaire, which did not differ between urban and rural areas or between tertiary and secondary hospitals (Table 3), indicating patients’ poor understanding of COPD.

**Table 3:** Baseline characteristics, COPD severity, and prescribed maintenance medications by residential area and by hospital tier

	Urban area (N = 2735)	Rural area (N = 2237)	Tertiary hospitals (N = 2597)	Secondary hospitals (N = 2381)
Severity of Airflow Limitation (GOLD stage), n/N (%)				
I	294/2524 (11.6)	163/1988 (8.2)	270/2337 (11.6)	188/2181 (8.6)
II	1127/2524 (44.7)	756/1988 (38.0)	1028/2337 (44.0)	858/2181 (39.3)
III	805/2524 (31.9)	751/1988 (37.8)	753/2337 (32.2)	805/2181 (36.9)
IV	298/2524 (11.8)	318/1988 (16.0)	286/2337 (12.2)	330/2181 (15.1)
GOLD 2016, n/N (%)				
A	397/2585 (15.4)	138/2108 (6.5)	395/2413 (16.4)	141/2286 (6.2)
B	602/2585 (23.3)	431/2108 (20.4)	603/2413 (25.0)	431/2286 (18.9)
C	338/2585 (13.1)	225/2108 (10.7)	299/2413 (12.4)	264/2286 (11.5)
D	1248/2585 (48.3)	1314/2108 (62.3)	1116/2413 (46.2)	1450/2286 (63.4)
GOLD 2017, n/N (%)				
A	574/2733 (21.0)	243/2237 (10.9)	602/2595 (23.2)	216/2381 (9.1)
B	1129/2733 (41.3)	951/2237 (42.5)	1173/2595 (45.2)	910/2381 (38.2)
C	212/2733 (7.8)	151/2237 (6.8)	155/2595 (6.0)	208/2381 (8.7)
D	818/2733 (29.9)	892/2237 (39.9)	665/2595 (25.6)	1047/2381 (44.0)
COPD-Q score,^a^ mean (SD)	5.9 (2.1)	6.0 (2.0)	5.8 (2.1)	6.1 (1.9)
Prescribed mono- or combination therapy maintenance therapies for COPD, n/N (%)				
ICS	3/2735 (0.1)	2/2237 (0.1)	2/2597 (0.1)	3/2381 (0.1)
LABA	22/2735 (0.8)	9/2237 (0.4)	9/2597 (0.3)	22/2381 (0.9)
ICS/LABA	619/2735 (22.6)	696/2237 (31.1)	682/2597 (26.3)	634/2381 (26.6)
SABA	34/2735 (1.2)	68/2237 (3.0)	11/2597 (0.4)	91/2381 (3.8)
SAMA	13/2735 (0.5)	14/2237 (0.6)	6/2597 (0.2)	21/2381 (0.9)
SABA/SAMA	1/2735 (0.0)	1/2237 (0.0)	2/2597 (0.1)	0
LAMA	500/2735 (18.3)	252/2237 (11.3)	479/2597 (18.4)	275/2381 (11.5)
ICS/LABA + LAMA	533/2735 (19.5)	336/2237 (15.0)	604/2597 (23.3)	267/2381 (11.2)
Methylxanthines	71/2735 (2.6)	133/2237 (5.9)	33/2597 (1.3)	171/2381 (7.2)
TCM	38/2735 (1.4)	42/2237 (1.9)	23/2597 (0.9)	57/2381 (2.4)

All percentages were calculated based on patients with available data

*COPD* chronic obstructive pulmonary disease, *COPD-Q* COPD knowledge questionnaire, *GOLD* global initiative for chronic obstructive lung disease, *ICS* inhaled corticosteroid, *LABA* long-acting beta_2_-agonist, *LAMA* long-acting muscarinic antagonist, *SABA* short-acting beta_2_-agonist, *SAMA* short-acting muscarinic antagonist, *TCM* traditional Chinese medicine

^a^COPD-Q scores were missing for three and two patients from urban and rural areas, and for two and three patients from tertiary and secondary hospitals, respectively

### Severity distribution

**Fig. 1:**
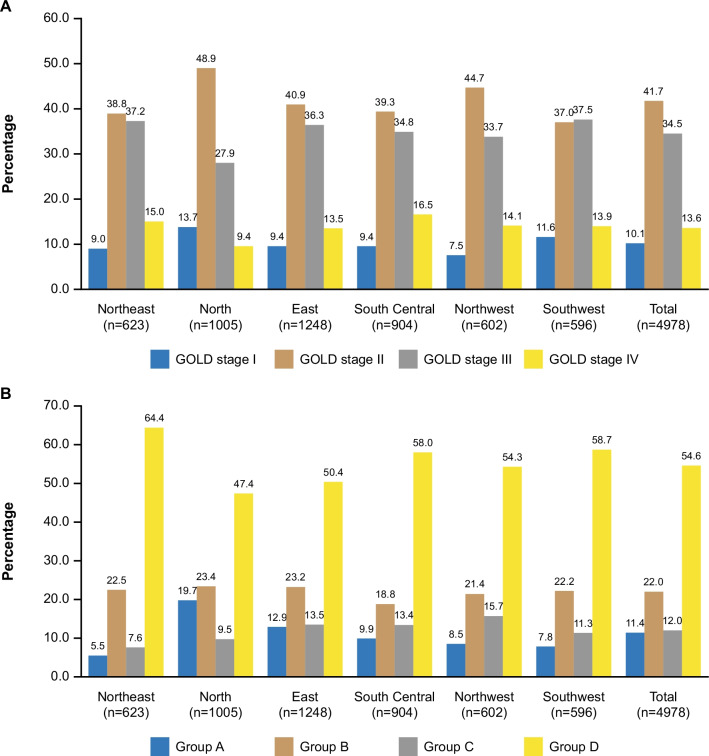
Severity distribution of COPD across six geographical regions in China. **A** Distribution of airway limitation stages. **B** Distribution of Group A‒D assessed as per GOLD 2016. Percentages of patients in each group are indicated. *COPD* chronic obstructive pulmonary disease, *GOLD* global initiative for chronic obstructive lung disease

Based on spirometry measurements, 458 (10.1%), 1886 (41.7%), 1558 (34.5%), and 616 (13.6%) patients were classified as GOLD stage I, II, III, and IV, respectively (Fig. 1A). Evaluated as per GOLD 2016 criteria, 536 (11.4%), 1034 (22.0%), 563 (12.0%), and 2566 (54.6%) patients were classified as Group A, B, C and D (Fig. 1B). Re-evaluated as per GOLD 2017 criteria, for the post-hoc analysis, Group B (2083 [41.9%]) constituted the largest group, followed by Group D (1712 [34.4%], see Additional file 1: Figure S1).

A comparison between CAT and mMRC as a symptom rater to classify GOLD groups showed that the symptom severity cutoff CAT ≥ 10 and breathless cutoff mMRC ≥ 2 had moderate agreement in categorising both GOLD 2016 and GOLD 2017 groups (Kappa coefficient, 0.481 across GOLD 2016 groups, 0.505 across GOLD 2017 groups, Table 4).

**Table 4:** Distribution of patients across GOLD groups using either CAT or mMRC as a symptom rater

		CAT ≥ 10				
	mMRC ≥ 2	A	B	C	D	Total
GOLD 2016	A	536 (11.4)	650 (13.8)	0	0	1186 (25.2)
	B	51 (1.1)	333 (7.1)	0	0	384 (8.2)
	C	0	0	563 (12.0)	921 (19.6)	1484 (31.6)
	D	0	0	142 (3.0)	1503 (32.0)	1645 (35.0)
	Total	587 (12.5)	983 (20.9)	705 (15.0)	2424 (51.6)	4699 (100.0)
	Kappa coefficient					0.481
GOLD 2017	A	818 (16.4)	1069 (21.5)	0	0	1887 (37.9)
	B	118 (2.4)	896 (18.0)	0	0	1014 (20.4)
	C	0	0	363 (7.3)	595 (12.0)	958 (19.3)
	D	0	0	85 (1.7)	1032 (20.7)	1117 (22.4)
	Total	936 (18.8)	1965 (39.5)	448 (9.0)	1627 (32.7)	4976 (100.0)
	Kappa coefficient					0.505

*CAT* COPD Assessment Test to assess symptoms, *COPD* chronic obstructive pulmonary disease, *GOLD* Global Initiative for Chronic Obstructive Lung Disease, *mMRC* modified Medical Research Council breathlessness assessment. Data are the number of patients (% patients)

Severity distribution between urban and rural areas was generally similar, with notable exceptions being a higher proportion of patients in GOLD Group A and a lower proportion of patients in Group D in urban than in rural areas and in tertiary hospitals than in secondary hospitals (with both GOLD 2016 and GOLD 2017 groups, Table 3).

More patients had a blood eosinophil count ≥ 300 cells/μL in Group A/C than in Group B/D (see Additional file 2: Figure S2).

### Pharmacological and non-pharmacological treatments

**Fig. 2:**
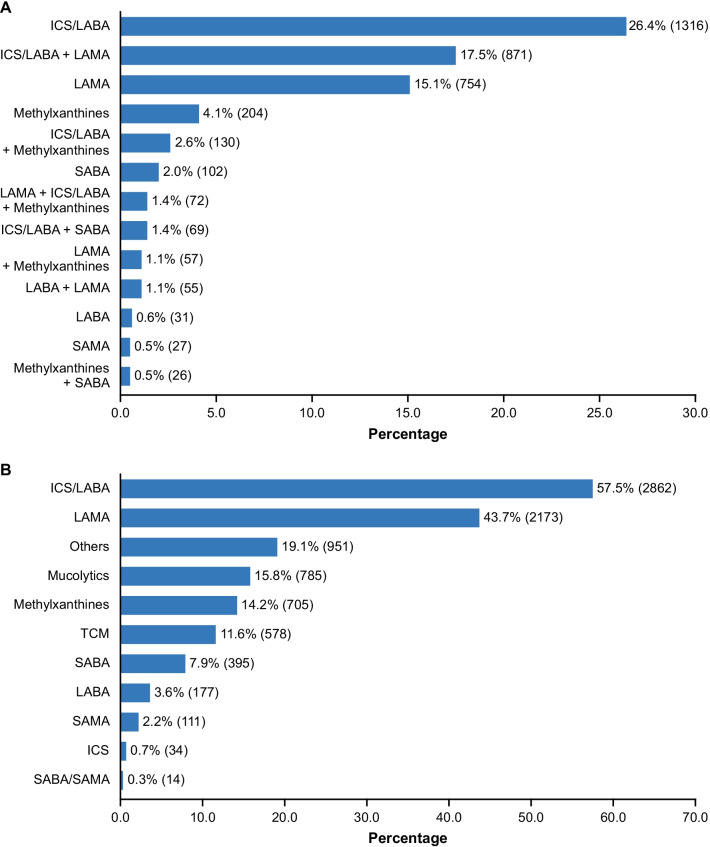
Distribution of maintenance medications for COPD. **A** Distribution of prescribed mono- and combination maintenance therapies for COPD. Mono- or combination therapies with bronchodilators and/or ICS (prescribed for ≥ 0.5% of patients) are shown, with no prohibition of use with mucolytics and other class of medications. **B** Distribution of medications prescribed in any form for COPD. Percentages and numbers of patients prescribed with each class of medications are indicated. *COPD* chronic obstructive pulmonary disease, *ICS* inhaled corticosteroid, *LABA* long-acting beta_2_-agonist, *LAMA* long-acting muscarinic antagonist, *SABA* short-acting beta_2_-agonist, *SAMA* short-acting muscarinic antagonist, *TCM* traditional Chinese medicine

Inhaled corticosteroids and long-acting beta_2_-agonist combinations (ICS/LABA, 1316 [26.4%]), ICS/LABA plus long-acting muscarinic antagonists (ICS/LABA + LAMA, 871 [17.5%]), and LAMA alone (754 [15.1%]) were the most commonly prescribed maintenance therapies (Fig. 2A and see Additional file 3: Table S1). However, many patients (681 [13.7%]) were not prescribed ICS or long-acting bronchodilators, the mainstay long-term inhaled medications, for symptom alleviation. Methylxanthines (705 [14.2%]) and mucolytics (785 [15.8%]) were also frequently prescribed (Fig. 2B and see Additional file 3: Table S1). Traditional Chinese medicine (TCM, 578 [11.6%]) and other medications, such as leukotriene inhibitors/ methoxyphenamine (non-recommended medications, were commonly prescribed (951 [19.1%]). Overall, ICS containing therapy was not prescribed based on blood eosinophil count: it was used by 67.7% of patients with a blood eosinophil count < 100 cells/μL, 62.1% of those with a blood eosinophil count ≥ 100 cells/μL and < 300 cells/μL, and 67.9% of those with a blood eosinophil count ≥ 300 cells/μL.

An increasing percentage of ICS/LABA + LAMA use was noted from GOLD stage I to IV, and its use was more common in GOLD 2016 Group C and D (see Additional file 3: Tables S1, S3). ICS/LABA, ICS/LABA + LAMA, and LAMA alone constituted the main therapies in all GOLD 2016 groups (22.4–29.1%, 13.4–19.9%, and 10.7–26.7%, respectively). Despite having more symptoms and/or higher risk of exacerbations, 156 (15.1%) patients in Group B, 57 (10.1%) in Group C, and 391 (15.2%) in Group D were not prescribed ICS or long-acting bronchodilators (see Additional file 3: Table S2). Reanalysis according to GOLD 2017 criteria revealed a consistent pattern and conclusion (see Additional file 3: Table S4, S5).

The preferences for classes of maintenance therapies prescribed did not vary much by regional location (see Additional file 3: Table S6). Regionally, highest incidence of non-recommended medication prescription, excluding TCM, occurred in the south-central region (310 [34.3%], see Additional file 3: Table S7).

Prescription of class of maintenance therapy was influenced by tier of hospital and rural/urban location (Table 3). LAMA monotherapy and ICS/LABA + LAMA were more commonly used whereas short-acting beta_2_-agonists (SABA) and methylxanthines were less commonly used in urban areas than in rural areas and by tertiary hospitals than by secondary hospitals. In addition, rural areas also used ICS/LABA more often than urban areas.

Non-pharmacological treatments for COPD included patient education (2976 [59.8%]), smoking cessation (3342 [67.1%]), breathing exercise (1567 [31.5%]), and vaccination (391 [7.9%], mainly for pneumonia [4.5%] and influenza [4.9%]).

## Discussion

This study provided an overview of the severity and management of COPD based on a sample of outpatients in China. Most Chinese patients with COPD belonged to GOLD stage II/III and GOLD 2016 Group D, without notable variations across regions in COPD severity distribution. ICS/LABA, ICS/LABA + LAMA, and LAMA were commonly prescribed as maintenance therapies for all severity groups and in all regions, and non-recommended medications were also frequently used. Location urban/rural status and tier of hospital did influence the choice of therapy, with triple combination therapy more commonly used in tertiary setting and urban locations. Our results highlighted high disease burden and unstandardized COPD treatment in China.

The sociodemographic profile of COPD patients depicted by our cohort is consistent with that in previous studies, which epitomises characteristics of the Chinese COPD population at large: a higher prevalence among men and the elderly, a high prevalence of smoke exposure [1, 2], and low levels of education in patients with COPD [2, 12]*.* These features are also recognised as risk factors for COPD [18]. Though having been diagnosed with COPD for a mean of 3.8 years, patients enrolled in our study still had inadequate knowledge of this disease, suggesting a need for patient education.

The proportion of exacerbation in this study was 49.4%, and higher than that reported by Fang et al. (5.9%) [1] but lower than that by Cui et al. (62.7%) [12]. The differences might reflect the respective study populations, Fang et al. [1] examined all COPD patients above 40 years from the integrated national disease surveillance points, while our study and Cui et al*.* [12] examined COPD outpatients above 40 years presenting at secondary or tertiary hospitals. Results from a population-based survey conducted in nine Asia–Pacific territories in 2012 revealed an exacerbation rate as high as 46% [19]. Exacerbations requiring hospitalisation or emergency room visits occurred at a rate of 0.5 and 0.2 per patient per year, respectively, indicating a high disease burden among Chinese outpatients with COPD and inadequacy in disease stabilisation and exacerbation prevention.

Published data on the distribution of COPD stages in China are conflicting, likely due to different diagnostic criteria and sampling strategies. Several population-based studies that collected data by on-site interview and physical examination and diagnosed COPD based on spirometry only characterised Chinese patients with COPD as being mostly mild in severity [2, 20]. The BOLD study estimated that about 85.1% of COPD patients in China belonged to GOLD stage I and II [20], while the China Pulmonary Health study reported an even higher proportion (GOLD stage I/II > 90%) [2]. Similarly, a nationwide survey found that GOLD stage I and II constituted 56.4% and 36.3%, respectively [1]. In contrast to previous reports [1, 2, 20], we showed that stage II (41.7%) and III (34.5%) were most prevalent. One possible explanation for this discrepancy is that early-stage patients who are asymptomatic or have tolerable symptoms may not seek medical advice and remain undiagnosed, leading to underrepresentation in our study. The discrepancy in the proportion of patients with early-stage COPD also suggests that strategies to improve early diagnosis, via education and screening, for instance, are needed in China. However, our data (54.6%) and other studies (43.0–70.7%) [12, 21] consistently showed predominance by Group D, who have more symptoms and high risk of exacerbations and mortality [22, 23]. Although a regional divide in COPD prevalence exists in China (highest prevalence reported in the Southwest region [20.2%] and lowest prevalence in Central China [10.2%]) [1], severity distribution did not appear to vary across geographical regions in our study. Increased prevalence in the Southwest region might be attributed to growing biofuel use and production from edible feedstocks such as *jatropha curcas*, and the consequential loss of natural area (forests) [24–26], as well as to increased indoor exposure to biofuel smoke [1, 27]. Furthermore, rural areas appear to have a higher disease burden based on a large proportion of Group D patients. Similar observations were reported in the US, with a higher prevalence rates and greater morbidity observed in the rural areas compared with urban areas [28, 29]. Results from an analysis of data from selected Asian countries from the Global Burden of Disease Study 2017 indicated that both socioeconomic and environmental factors impact COPD mortality rates [30, 31]. The increased disease burden in rural areas may be associated with ageing population, increased smoking prevalence, obstacles to care, such as less access to early diagnosis and treatment, suboptimal disease management, poor disease awareness among patients and doctors, and differences in lifestyle [32–34]. Special attention should be afforded to lifestyle, given that some social behaviours banned in the cities occur quite frequently in less-developed rural areas, emphasizing that the concept of ecological civilisation needs to be improved [33]. Additionally, the differences in COPD severity distribution between urban and rural areas might reflect different exposure to environmental recognized risk factors for COPD, such as occupational dust and chemicals and indoor air pollution [1, 27, 32, 34]. Excessive use of solid biofuel (such as wood) for heating and cooking purposes and unventilated households contribute to indoor air pollution and were identified as potential contributing factors for greater disease burden in rural areas.

A change in the combined assessment system was stipulated by GOLD 2017 Report, in which airflow limitation no longer factors into the assessment [10] based on findings that it does not help improve prediction of exacerbation or mortality [35, 36]. It remains unclear whether GOLD 2017 criteria outperforms GOLD 2016 in stratifying patients and guiding treatment decisions. Both frameworks classify patients into clinically homogeneous groups [22, 23, 37]. Switching from GOLD 2016 to GOLD 2017 groups led to a redistribution of patients among different groups in our study, especially reducing the proportion of Group D whilst increasing that of Group B, consistent with the ‘vertical’ shift from D to B anticipated and observed in previous reports [12, 21, 38–40]. Follow-up on these patients’ disease progression and treatment outcomes will provide real-world evidence for comparing two systems.

Although the results on maintenance medications do not allow a precise estimate of adherence/nonadherence to GOLD strategy document due to no differentiation between initial and subsequent treatments, they revealed widely prescribed medications for COPD maintenance in China. Overall, long-acting bronchodilators were more widely used than short-acting bronchodilators, which is in line with current guidelines [9]. However, notable deviations from guidelines were observed [9, 17]. Dual ICS/LABA and triple ICS/LABA + LAMA therapy seemed to be overused, especially for Group A and B, which was also shown by another Chinese study [12]. In addition, results from the Taiwan obstructive lung disease (TOLD) study revealed that about one-third of Group A/B patients are prescribed with ICS-containing therapy [41]. In another study based on data from the Swedish National Airway Register (SNAR), about 33% of Group A and 46% of Group B patients were treated with ICS, suggesting a high use of ICS containing therapy [42]. In order to optimize the treatment in patients with COPD, factors such as high blood eosinophil counts or fractional exhaled nitric oxide (FeNO) levels, a past medical history of asthma or allergic rhinitis or findings suggestive of asthma-COPD overlap (ACO) should be considered to identify patients who would benefit from ICS use [10, 43, 44]. The GOLD 2017 Report proposed a stepwise approach to ICS overprescription based on evidence from FLAME and WISDOM studies, with dose escalation and de-escalation strategies according to the individual patient symptoms and exacerbation risk [10]. Triple therapy was used at a slightly higher rate in GOLD stage III/IV and GOLD 2016 Group C/D but not in GOLD 2017 Group C/D, indicating that the prescription was based on severe/very severe airflow limitation [38], probably with an aim to relieve symptoms in these patients. In addition, the current lack of access to dual bronchodilators in China might result in the low rate of LABA + LAMA prescription. Alarmingly, a great number of patients in each GOLD group were on ICS/LABA + LAMA, although there is a possibility of stepping up or down treatment based on patient response. ICS might be preferred by physicians as an add-on for exacerbation prevention, especially for patients at high risk. In addition, given that patients with concurrent asthma only accounted for 8.8%, the widespread use of ICS/LABA was unlikely to be due to this concomitant condition. Mucolytics were the third most frequently prescribed class of medications (15.8%), which might help relieve symptoms and improve quality of life [45–47]. Non-recommended medications were commonly prescribed, an issue also highlighted by Fang et al. (TCM, 16.3%; antibiotics, 60.5%) [14] and by Ding et al. (16.5%) [15]. In summary, treatment adherence to GOLD strategy document needs to be improved, and this can only be achieved by improving both physicians’ adherence to guidelines, so that the adequate treatment is prescribed, and patient’s adherence to physician’s advice and to the prescribed medication. Patient’s understanding of COPD and the complexity of the prescribed treatment are critical factors. Therefore, improved patient education may increase adherence rates and ultimately improve patient outcomes [48–50].

GOLD 2019 Report recommends the use of blood eosinophil count as a biomarker to guide follow-up treatment with ICS, with ≥ 300 cells/μL favouring ICS prescription while < 100 cells/μL opposing the use of ICS [51]. Although prescriptions of ICS therapies did not seem to be based on blood eosinophil counts in our patient population, our longitudinal data on treatment response provides further evidence on the use of higher blood eosinophil counts to predict better response to ICS therapy.

Regional inequality in infrastructure and qualified personnel remains an obstacle to delivering high-quality health care to most Chinese residents [52]. Specialised respiratory departments are instituted mainly in secondary and tertiary hospitals, but substantial disparities exist in the capability, expertise, and performance of respiratory care between hospitals of these two tiers and in different regions [53]. Investments and efforts to improve health care should be tilted towards the rural regions and secondary hospitals, which may help address the disparities in COPD burden revealed in our study.

This study has several limitations. First, only patients who visited the outpatient respiratory department of secondary and tertiary hospitals who were diagnosed with COPD were enrolled. Given the previously reported serious issue of underdiagnosis [1] and aforementioned inconsistency in the proportions of GOLD stages characterised by our study and others [1, 2, 20], it is likely that a fraction of patients with COPD, especially those at early stages and with few or mild symptoms, were not represented by the study. Also, patients at GOLD stage 0, a controversial category usually defined as the presence of COPD symptoms without airflow obstruction [54], were excluded by the criterion of FEV_1_/FVC < 70% in this study. Thus, caution should be taken when extrapolating the results from the outpatients in this study to the general patient population. Baseline data on maintenance medications were collected retrospectively and included both initial and follow-up treatments; therefore, non-adherence to GOLD strategy document cannot be precisely estimated. Baseline data on symptoms and exacerbations were recalled retrospectively by patients, possibly resulting in potential incompleteness. Finally, data were confined to results of examinations and tests performed in routine clinical practice, and therefore not all data were available for each patient (e.g., blood tests, fractional exhaled nitric oxide tests, or chest computed tomography scans).

To improve patient outcomes, these data indicate that focussed and widespread physician education on which classes of COPD treatment at which stages give the most effective outcomes would be beneficial, as would an increased implementation of patient education and vaccination against influenza and pneumonia for patients at risk of exacerbations with COPD.

## Conclusions

Moderate-to-severe airflow obstruction, more symptoms, and high risk of exacerbations are prevalent among patients diagnosed with COPD in China. Maintenance therapies are not prescribed with full adherence to guideline recommendations.

## Supplementary Information

**Additional file 1: Figure S1.** Distribution of Group A‒D assessed as per GOLD 2017. Percentages of patients in each group are indicated. GOLD, Global Initiative for Chronic Obstructive Lung Disease.

**Additional file 2: Figure S2.** Percentages of patients with different levels of blood eosinophil counts in GOLD Group A‒D. GOLD groups classified according to GOLD 2016 (the second row) or GOLD 2017 (the third row). Number and percentage of patients with valid blood eosinophil counts in each GOLD group are provided in each cell below the pie chart. EOS, blood eosinophil count; GOLD, Global Initiative for Chronic Obstructive Lung Disease.

**Additional file 3: Table S1.** Distribution of prescribed mono- and combination maintenance therapies for COPD by severity group (GOLD 2016). **Table S2.** Distribution of medications prescribed in any form for COPD maintenance by severity group (GOLD 2016). **Table S3.** Distribution of prescribed stable COPD medications as drug class in mono- or combination therapies by baseline COPD airway limitation severity. **Table S4.** Distribution of prescribed mono- and combination maintenance therapies for COPD by severity group (GOLD 2017). **Table S5.** Distribution of medications prescribed in any form for COPD maintenance by severity group (GOLD 2017). **Table S6.** Distribution of prescribed mono- and combination maintenance therapies for COPD by geographical regions. **Table S7.** Distribution of medications prescribed in any form for COPD maintenance by geographical regions.

## Data Availability

The datasets used and/or analysed during the current study are available from the corresponding author on reasonable request.

## Abbreviations

BOLD: Burden of obstructive lung disease
CAT: COPD assessment test
COPD: Chronic obstructive pulmonary disease
EOS: Blood eosinophil count
FAS: Full analysis set
FEV1: Forced expiratory volume in one second
FVC: Forced vital capacity
GOLD: Global initiative for chronic obstructive lung disease
ICF: Informed consent form
ICS: Inhaled corticosteroid
LABA: Long-acting β2 agonist
LAMA: Long-acting anti-muscarinic agent
mMRC: Modified Medical Research Council dyspnoea scale
SABA: Short-acting β2 agonist
SAMA: Short-acting anti-muscarinic agent
SAS: Statistical analysis system
SD: Standard deviation
TCM: Traditional Chinese medicine
USD: United States dollar
