# Correction to: Severity distribution and treatment of chronic obstructive pulmonary disease in China: baseline results of an observational study

**DOI:** 10.1186/s12931-022-02068-9

**Published:** 2022-06-18

**Authors:** Ting Yang, Baiqiang Cai, Bin Cao, Jian Kang, Fuqiang Wen, Yahong Chen, Wenhua Jian, Hongyan Shang, Chen Wang

**Affiliations:** 1 Department of Pulmonary and Critical Care Medicine, China-Japan Friendship Hospital, Beijing, 100029 China; 2 National Clinical Research Center for Respiratory Diseases, Beijing, 100029 China; 3 Department of Respiratory and Critical Care Medicine, Peking Union Medical College Hospital, Beijing, China; 4 Department of Respiratory and Critical Care Medicine, The First Hospital of China Medical University, Shenyang, China; 5 Department of Respiratory and Critical Care Medicine, West China Hospital, Sichuan University, Chengdu, China; 6 Department of Respiratory and Critical Care Medicine, Peking University Third Hospital, Beijing, China; 7 State Key Laboratory of Respiratory Disease, Guangzhou Institute of Respiratory Disease, National Clinical Research Center for Respiratory Disease, 1st Affiliated Hospital of Guangzhou Medical University, Guangzhou, China; 8 Department of Medical Affairs, AstraZeneca China, Shanghai, China

## Correction to: Respiratory Research (2022) 23:106 10.1186/s12931-022-02021-w

Following the publication of the original article [1], the authors identified errors in the study enrolment dates in the Abstract and Results sections. The dates “15 December 2017 and 6 August 2020” should be corrected to “30 June 2017 and 29 January 2019”.

The original article has been corrected.
