# Supplementary figures and images for: Severity distribution and treatment of chronic obstructive pulmonary disease in China: baseline results of an observational study
Source: Respir Res. 2022 Apr 29;23:106. doi: 10.1186/s12931-022-02021-w (PMC9052685; doi:10.1186/s12931-022-02021-w)

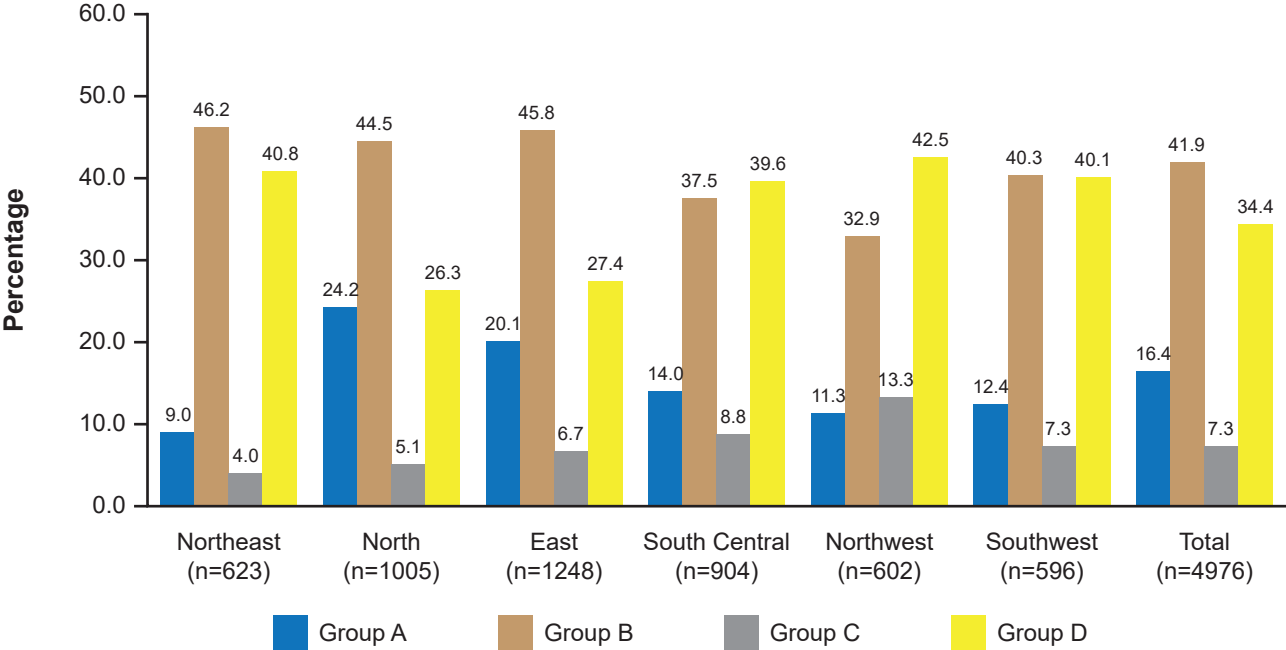

Supplement: Supplementary file 1 — Additional file 1: Figure S1. Distribution of Group A‒D assessed as per GOLD 2017. Percentages of patients in each group are indicated. GOLD, Global Initiative for Chronic Obstructive Lung Disease. [file 12931_2022_2021_MOESM1_ESM.pdf]

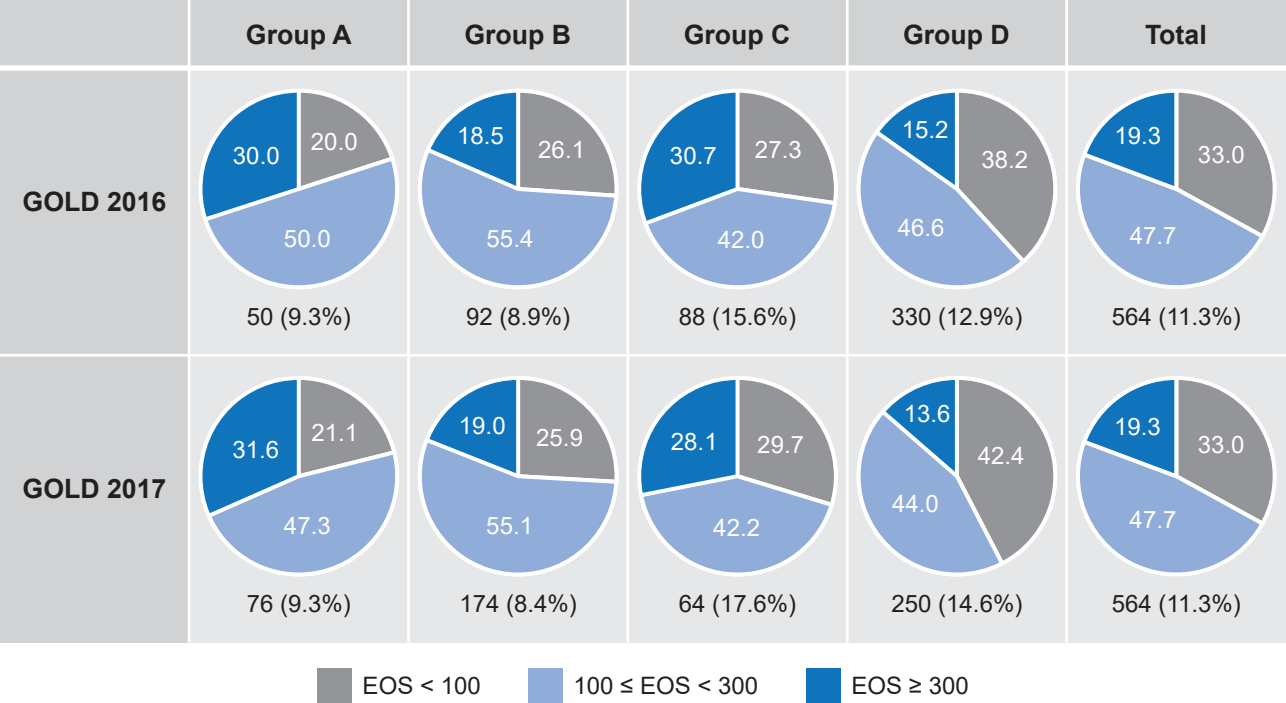

Supplement: Supplementary file 2 — Additional file 2: Figure S2. Percentages of patients with different levels of blood eosinophil counts in GOLD Group A‒D. GOLD groups classified according to GOLD 2016 (the second row) or GOLD 2017 (the third row). Number and percentage of patients with valid blood eosinophil counts in each GOLD group are provided in each cell below the pie chart. EOS, blood eosinophil count; GOLD, Global Initiative for Chronic Obstructive Lung Disease. [file 12931_2022_2021_MOESM2_ESM.pdf]
