# Supplementary material for: Severity distribution and treatment of chronic obstructive pulmonary disease in China: baseline results of an observational study
Source: Respir Res. 2022 Apr 29;23:106. doi: 10.1186/s12931-022-02021-w (PMC9052685; doi:10.1186/s12931-022-02021-w)
Supplement: Supplementary file 3 — Additional file 3: Table S1. Distribution of prescribed mono- and combination maintenance therapies for COPD by severity group (GOLD 2016). Table S2. Distribution of medications prescribed in any form for COPD maintenance by severity group (GOLD 2016). Table S3. Distribution of prescribed stable COPD medications as drug class in mono- or combination therapies by baseline COPD airway limitation severity. Table S4. Distribution of prescribed mono- and combination maintenance therapies for COPD by severity group (GOLD 2017). Table S5. Distribution of medications prescribed in any form for COPD maintenance by severity group (GOLD 2017). Table S6. Distribution of prescribed mono- and combination maintenance therapies for COPD by geographical regions. Table S7. Distribution of medications prescribed in any form for COPD maintenance by geographical regions. [file 12931_2022_2021_MOESM3_ESM.docx]

**Severity distribution and treatment of chronic obstructive pulmonary disease in China: baseline results of an observational study**

Ting Yang,^1^ MD, Baiqiang Cai,^2^ MMed, Bin Cao,^1^ MD, Jian Kang,^3^ MD, Fuqiang Wen,^4^ MD, PhD, Yahong Chen,^5^ MD, Wenhua Jian,^6^ MD, Hongyan Shang,^7^ MMed, and Chen Wang,^1,*^ MD

*^1^Department of Pulmonary and Critical Care Medicine, China-Japan Friendship Hospital; National Clinical Research Center for Respiratory Diseases; Institute of Respiratory Medicine, Chinese Academy of Medical Science, Beijing, China; ^2^Department of Respiratory and Critical Care Medicine, Peking Union Medical College Hospital, Beijing, China; ^3^Department of Respiratory and Critical Care Medicine, The First Hospital of China Medical University, Shenyang, China; ^4^Department of Respiratory and Critical Care Medicine, West China Hospital, Sichuan University, Chengdu, China; ^5^Department of Respiratory and Critical Care Medicine, Peking University Third Hospital, Beijing, China; ^6^State Key Laboratory of Respiratory Disease, Guangzhou Institute of Respiratory Disease, National Clinical Research Center for Respiratory Disease, 1st Affiliated Hospital of Guangzhou Medical University, Guangzhou, China; ^7^Department of Medical Affairs, AstraZeneca China, Shanghai, China*.

***Corresponding author**: Professor Chen Wang, Department of Pulmonary and Critical Care Medicine, China-Japan Friendship Hospital; National Clinical Research Center for Respiratory Diseases; Institute of Respiratory Medicine, Chinese Academy of Medical Science, No 2, East Yinghua Road, Chaoyang District, Beijing 100029, China; Tel: 86-10-68792233; Fax: 86-10-68792233; [wangchen66366@163.com](mailto:wangchen66366@163.com)

[**Table E1. Distribution of prescribed mono- and combination maintenance therapies for COPD by severity group (GOLD 2016)** 3](#_Toc62486097)

[**Table E2. Distribution of medications prescribed in any form for COPD maintenance by severity group (GOLD 2016)** 5](#_Toc62486098)

[**Table E3. Distribution of prescribed stable COPD medications as drug class in mono- or combination therapies by baseline COPD airway limitation severity** 6](#_Toc62486099)

[**Table E4. Distribution of prescribed mono- and combination maintenance therapies for COPD by severity group (GOLD 2017)** 8](#_Toc62486100)

[**Table E5. Distribution of medications prescribed in any form for COPD maintenance by severity group (GOLD 2017)** 10](#_Toc62486101)

[**Table E6. Distribution of prescribed mono- and combination maintenance therapies for COPD by geographical regions** 11](#_Toc62486102)

[**Table E7. Distribution of medications prescribed in any form for COPD maintenance by geographical regions** 13](#_Toc62486103)

**Table E1. Distribution of prescribed mono- and combination maintenance therapies for COPD by severity group (GOLD 2016)**

| Mono-  or combination therapy,^*^ n (%) | Group A  (n = 536) | Group B  (n = 1034) | Group C  (n = 563) | Group D  (n = 2566) | Total  (N = 4978) |
| --- | --- | --- | --- | --- | --- |
| ICS | 1 (0.2) | 1 (0.1) | 0 | 0 | 5 (0.1) |
| LABA | 4 (0.7) | 7 (0.7) | 1 (0.2) | 19 (0.7) | 31 (0.6) |
| ICS/LABA | 120 (22.4) | 245 (23.7) | 164 (29.1) | 685 (26.7) | 1316 (26.4) |
| SABA | 5 (0.9) | 17 (1.6) | 7 (1.2) | 68 (2.7) | 102 (2.0) |
| SAMA | 3 (0.6) | 1 (0.1) | 7 (1.2) | 15 (0.6) | 27 (0.5) |
| SABA/SAMA | 1 (0.2) | 0 | 0 | 1 (0.0) | 2 (0.0) |
| LAMA | 143 (26.7) | 213 (20.6) | 85 (15.1) | 275 (10.7) | 754 (15.1) |
| Methylxanthines | 17 (3.2) | 65 (6.3) | 12 (2.1) | 107 (4.2) | 204 (4.1) |
| ICS/LABA + LAMA | 81 (15.1) | 139 (13.4) | 112 (19.9) | 489 (19.1) | 871 (17.5) |
| LABA + LAMA | 4 (0.7) | 13 (1.3) | 6 (1.1) | 32 (1.2) | 55 (1.1) |
| LAMA + ICS | 0 | 0 | 0 | 1 (0.0) | 2 (0.0) |
| LAMA + SABA | 2 (0.4) | 5 (0.5) | 1 (0.2) | 14 (0.5) | 22 (0.4) |
| LAMA + Methylxanthines | 13 (2.4) | 13 (1.3) | 6 (1.1) | 23 (0.9) | 57 (1.1) |
| ICS/LABA + SABA | 1 (0.2) | 8 (0.8) | 9 (1.6) | 50 (1.9) | 69 (1.4) |
| ICS/LABA + Methylxanthines | 12 (2.2) | 26 (2.5) | 19 (3.4) | 70 (2.7) | 130 (2.6) |
| LAMA + ICS/LABA + SABA | 1 (0.2) | 0 | 3 (0.5) | 16 (0.6) | 21 (0.4) |
| LAMA + ICS/LABA + Methylxanthines | 8 (1.5) | 11 (1.1) | 6 (1.1) | 45 (1.8) | 72 (1.4) |
| LAMA + LABA + ICS | 0 | 0 | 0 | 0 | 0 |
| LAMA + LABA + SABA | 0 | 0 | 0 | 0 | 0 |
| LAMA + LABA + Methylxanthines | 0 | 2 (0.2) | 1 (0.2) | 3 (0.1) | 6 (0.1) |
| Methylxanthines + SABA | 2 (0.4) | 3 (0.3) | 0 | 19 (0.7) | 25 (0.5) |

^*^ Subjects prescribed one and only mono- or combination of medications on display, with no prohibition of being prescribed mucolytic, traditional Chinese medicine and others at the same time.

COPD, chronic obstructive pulmonary disease; GOLD, Global Initiative for Chronic Obstructive Lung Disease; ICS, inhaled corticosteroid; LABA, long-acting beta_2_-agonist; LAMA, long-acting muscarinic antagonist; SABA, short-acting beta_2_-agonist; SAMA, short-acting muscarinic antagonist.

**Table E2. Distribution of medications prescribed in any form for COPD maintenance by severity group (GOLD 2016)**

| Medications used in any form,^*^ n (%) | Group A  (n = 536) | Group B  (n = 1034) | Group C  (n = 563) | Group D  (n = 2566) | Total  (N = 4978) |
| --- | --- | --- | --- | --- | --- |
| ICS | 3 (0.6) | 5 (0.5) | 1 (0.2) | 19 (0.7) | 34 (0.7) |
| LABA | 13 (2.4) | 42 (4.1) | 14 (2.5) | 106 (4.1) | 177 (3.6) |
| ICS/LABA | 255 (47.6) | 516 (49.9) | 351 (62.3) | 1561 (60.8) | 2862 (57.5) |
| SABA | 13 (2.4) | 55 (5.3) | 29 (5.2) | 285 (11.1) | 395 (7.9) |
| SAMA | 9 (1.7) | 10 (1.0) | 17 (3.0) | 72 (2.8) | 111 (2.2) |
| SABA/SAMA | 3 (0.6) | 2 (0.2) | 0 | 9 (0.4) | 14 (0.3) |
| LAMA | 286 (53.4) | 489 (47.3) | 241 (42.8) | 1044 (40.7) | 2173 (43.7) |
| Methylxanthines | 60 (11.2) | 158 (15.3) | 56 (9.9) | 416 (16.2) | 705 (14.2) |
| Mucolytics | 92 (17.2) | 184 (17.8) | 91 (16.2) | 380 (14.8) | 785 (15.8) |
| TCM | 53 (9.9) | 156 (15.1) | 39 (6.9) | 295 (11.5) | 578 (11.6) |
| Others | 116 (21.6) | 224 (21.7) | 102 (18.1) | 457 (17.8) | 951 (19.1) |
| Neither ICS nor long-acting bronchodilator^†^ | 53 (9.0) | 156 (15.1) | 57 (10.1) | 391 (15.2) | 681 (13.7) |

^*^ Medications classified by drug class; ^†^ this class denotes prescriptions without ICS, LABA, ICS/LABA or LAMA.

COPD, chronic obstructive pulmonary disease; GOLD, Global Initiative for Chronic Obstructive Lung Disease; ICS, inhaled corticosteroid; LABA, long-acting beta_2_-agonist; LAMA, long-acting muscarinic antagonist; SABA, short-acting beta_2_-agonist; SAMA, short-acting muscarinic antagonist; TCM, traditional Chinese medicine.

**Table E3. Distribution of prescribed stable COPD medications as drug class in mono- or combination therapies by baseline COPD airway limitation severit****y**

| **Mono-**  **or combination therapy,^*^ n (%)** | **GOLD stage I (N=458)** | **GOLD stage** **II  (N=1886)** | **GOLD stage III (N=1558)** | **GOLD stage IV (N=616)** | **Total (N=4978)** |
| --- | --- | --- | --- | --- | --- |
| **ICS** | 0 | 2 (0.1) | 0 | 0 | 5 (0.1) |
| **LABA** | 1 (0.2) | 17 (0.9) | 7 (0.4) | 5 (0.8) | 31 (0.6) |
| **ICS/LABA** | 117 (25.5) | 498 (26.4) | 393 (25.2) | 138 (22.4) | 1316 (26.4) |
| **SABA** | 9 (2.0) | 34 (1.8) | 37 (2.4) | 8 (1.3) | 102 (2.0) |
| **SAMA** | 2 (0.4) | 17 (0.9) | 5 (0.3) | 2 (0.3) | 27 (0.5) |
| **SABA/SAMA** | 0 | 1 (0.1) | 1 (0.1) | 0 | 2 (0.0) |
| **LAMA** | 110 (24.0) | 374 (19.8) | 166 (10.7) | 53 (8.6) | 755 (15.1) |
| **Methylxanthines** | 29 (6.3) | 72 (3.8) | 71 (4.6) | 26 (4.2) | 204 (4.1) |
| **ICS/LABA + LAMA** | 47 (10.3) | 278 (14.7) | 313 (20.1) | 152 (24.7) | 871 (17.5) |
| **LABA + LAMA** | 3 (0.7) | 21 (1.1) | 25 (1.6) | 5 (0.8) | 55 (1.1) |
| **LAMA + ICS** | 0 | 0 | 0 | 1 (0.2) | 2 (0.0) |
| **LAMA + SABA** | 1 (0.2) | 9 (0.5) | 7 (0.4) | 5 (0.8) | 22 (0.4) |
| **LAMA + Methylxanthines** | 5 (1.1) | 23 (1.2) | 18 (1.2) | 7 (1.1) | 57 (1.1) |
| **ICS/LABA + SABA** | 5 (1.1) | 18 (1.0) | 29 (1.9) | 14 (2.3) | 69 (1.4) |
| **ICS/LABA + Methylxanthines** | 7 (1.5) | 51 (2.7) | 43 (2.8) | 23 (3.7) | 130 (2.6) |
| **LAMA + ICS/LABA + SABA** | 1 (0.2) | 6 (0.3) | 8 (0.5) | 2 (0.3) | 21 (0.4) |
| **LAMA + ICS/LABA + Methylxanthines** | 5 (1.1) | 22 (1.2) | 26 (1.7) | 15 (2.4) | 72 (1.4) |
| **LAMA + LABA + ICS** | 0 | 0 | 0 | 0 | 0 |
| **LAMA + LABA + SABA** | 0 | 0 | 0 | 0 | 0 |
| **LAMA + LABA + Methylxanthines** | 1 (0.2) | 1 (0.1) | 2 (0.1) | 2 (0.3) | 6 (0.1) |
| **Methylxanthines + SABA** | 2 (0.4) | 6 (0.3) | 7 (0.4) | 6 (1.0) | 26 (0.5) |

^*^ Subjects prescribed one and only mono- or combination of medications on display, with no prohibition of being prescribed mucolytic, traditional Chinese medicine and others at the same time.

COPD, chronic obstructive pulmonary disease; ICS, inhaled corticosteroid; LABA, long-acting beta_2_-agonist; LAMA, long-acting muscarinic antagonist; SABA, short-acting beta_2_-agonist; SAMA, short-acting muscarinic antagonist.

**Table E4. Distribution of prescribed mono- and combination maintenance therapies for COPD by severity group (GOLD 2017)**

| Mono-  or combination therapy,^*^ n (%) | Group A  (n = 818) | Group B  (n = 2083) | Group C  (n = 363) | Group D  (n = 1712) | Total  (N = 4978) |
| --- | --- | --- | --- | --- | --- |
| ICS | 1 (0.1) | 4 (0.2) | 0 | 0 | 5 (0.1) |
| LABA | 4 (0.5) | 13 (0.6) | 1 (0.3) | 13 (0.8) | 31 (0.6) |
| ICS/LABA | 198 (24.2) | 497 (23.9) | 119 (32.8) | 502 (29.3) | 1316 (26.4) |
| SABA | 7 (0.9) | 35 (1.7) | 7 (1.9) | 53 (3.1) | 102 (2.0) |
| SAMA | 4 (0.5) | 2 (0.1) | 7 (1.9) | 14 (0.8) | 27 (0.5) |
| SABA/SAMA | 1 (0.1) | 1 (0.1) | 0 | 0 | 2 (0.0) |
| LAMA | 185 (22.6) | 322 (15.5) | 58 (16.0) | 188 (11.0) | 754 (15.1) |
| Methylxanthines | 25 (3.1) | 122 (5.9) | 5 (1.4) | 52 (3.0) | 204 (4.1) |
| ICS/LABA + LAMA | 150 (18.3) | 354 (17.0) | 57 (15.7) | 309 (18.0) | 871 (17.5) |
| LABA + LAMA | 6 (0.7) | 30 (1.4) | 4 (1.1) | 15 (0.9) | 55 (1.1) |
| LAMA + ICS | 0 | 2 (0.1) | 0 | 0 | 2 (0.0) |
| LAMA + SABA | 3 (0.4) | 8 (0.4) | 0 | 11 (0.6) | 22 (0.4) |
| LAMA + Methylxanthines | 16 (2.0) | 29 (1.4) | 3 (0.8) | 9 (0.5) | 57 (1.1) |
| ICS/LABA + SABA | 2 (0.2) | 18 (0.9) | 8 (2.2) | 41 (2.4) | 69 (1.4) |
| ICS/LABA + Methylxanthines | 25 (3.1) | 48 (2.3) | 7 (1.9) | 50 (2.9) | 130 (2.6) |
| LAMA + ICS/LABA + SABA | 4 (0.5) | 5 (0.2) | 0 | 12 (0.7) | 21 (0.4) |
| LAMA + ICS/LABA + Methylxanthines | 11 (1.3) | 37 (1.8) | 3 (0.8) | 21 (1.2) | 72 (1.4) |
| LAMA + LABA + ICS | 0 | 0 | 0 | 0 | 0 |
| LAMA + LABA + SABA | 0 | 0 | 0 | 0 | 0 |
| LAMA + LABA + Methylxanthines | 1 (0.1) | 4 (0.2) | 0 | 1 (0.1) | 6 (0.1) |
| Methylxanthines + SABA | 2 (0.2) | 8 (0.4) | 0 | 16 (0.9) | 26 (0.5) |

^*^ Subjects prescribed one and only mono- or combination of medications on display, with no prohibition of being prescribed mucolytic, traditional Chinese medicine and others at the same time.

COPD, chronic obstructive pulmonary disease; GOLD, Global Initiative for Chronic Obstructive Lung Disease; ICS, inhaled corticosteroid; LABA, long-acting beta_2_-agonist; LAMA, long-acting muscarinic antagonist; SABA, short-acting beta_2_-agonist; SAMA, short-acting muscarinic antagonist.

**Table E5. Distribution of medications prescribed in any form for COPD maintenance by severity group (GOLD 2017)**

| Medications used in any form,^*^ n (%) | Group A  (n = 818) | Group B  (n = 2083) | Group C  (n = 363) | Group D  (n = 1712) | Total  (N = 4978) |
| --- | --- | --- | --- | --- | --- |
| ICS | 3 (0.4) | 15 (0.7) | 1 (0.3) | 15 (0.9) | 34 (0.7) |
| LABA | 17 (2.1) | 88 (4.2) | 10 (2.8) | 62 (3.6) | 177 (3.6) |
| ICS/LABA | 440 (53.8) | 1131 (54.3) | 220 (60.6) | 1070 (62.5) | 2862 (57.5) |
| SABA | 21 (2.6) | 141 (6.8) | 24 (6.6) | 209 (12.2) | 395 (7.9) |
| SAMA | 14 (1.7) | 22 (1.1) | 14 (3.9) | 61 (3.6) | 111 (2.2) |
| SABA/SAMA | 3 (0.4) | 5 (0.2) | 0 | 6 (0.4) | 14 (0.3) |
| LAMA | 424 (51.8) | 972 (46.7) | 135 (37.2) | 640 (37.5) | 2173 (43.7) |
| Methylxanthines | 91 (11.1) | 338 (16.2) | 28 (7.7) | 248 (14.5) | 705 (14.2) |
| Mucolytics | 147 (18.0) | 374 (18.0) | 47 (12.9) | 217 (12.7) | 785 (15.8) |
| TCM | 79 (9.7) | 313 (15.0) | 20 (5.5) | 166 (9.7) | 578 (11.6) |
| Others | 166 (20.3) | 445 (21.4) | 62 (17.1) | 277 (16.2) | 951 (19.1) |
| Neither ICS nor long-acting bronchodilator^†^ | 75 (9.2) | 321 (15.4) | 41 (11.3) | 244 (14.3) | 681 (13.7) |

^*^ Medications classified by drug class; ^†^ this class denotes prescriptions without ICS, LABA, ICS/LABA or LAMA.

COPD, chronic obstructive pulmonary disease; GOLD, Global Initiative for Chronic Obstructive Lung Disease; ICS, inhaled corticosteroid; LABA, long-acting beta_2_-agonist; LAMA, long-acting muscarinic antagonist; SABA, short-acting beta_2_-agonist; SAMA, short-acting muscarinic antagonist; TCM, traditional Chinese medicine.

**Table E6. Distribution of prescribed mono- and combination maintenance therapies for COPD by geographical regions**

| Mono-  or combination therapy,^*^  n (%) | Northeast  (n = 623) | North  (n = 1005) | East  (n = 1248) | South  Central  (n = 904) | Northwest  (n = 602) | Southwest  (n = 596) | Total  (N = 4978) |
| --- | --- | --- | --- | --- | --- | --- | --- |
| ICS | 0 | 3 (0.3) | 0 | 0 | 0 | 2 (0.3) | 5 (0.1) |
| LABA | 1 (0.2) | 2 (0.2) | 17 (1.4) | 8 (0.9) | 0 | 3 (0.5) | 31 (0.6) |
| ICS/LABA | 195 (31.3) | 247 (24.6) | 318 (25.5) | 231 (25.6) | 211 (35.0) | 114 (19.1) | 1316 (26.4) |
| SABA | 11 (1.8) | 34 (3.4) | 3 (0.2) | 33 (3.7) | 17 (2.8) | 4 (0.7) | 102 (2.0) |
| SAMA | 0 | 15 (1.5) | 5 (0.4) | 6 (0.7) | 1 (0.2) | 0 | 27 (0.5) |
| SABA/SAMA | 1 (0.2) | 0 | 0 | 0 | 1 (0.2) | 0 | 2 (0.0) |
| LAMA | 86 (13.8) | 146 (14.5) | 297 (23.8) | 76 (8.4) | 93 (15.4) | 56 (9.4) | 754 (15.1) |
| Methylxanthines | 105 (16.9) | 10 (1.0) | 5 (0.4) | 67 (7.4) | 3 (0.5) | 14 (2.3) | 204 (4.1) |
| ICS/LABA + LAMA | 101 (16.2) | 203 (20.2) | 310 (24.8) | 62 (6.9) | 99 (16.4) | 96 (16.1) | 871 (17.5) |
| LABA + LAMA | 1 (0.2) | 6 (0.6) | 41 (3.3) | 1 (0.1) | 0 | 6 (1.0) | 55 (1.1) |
| LAMA + ICS | 1 (0.2) | 1 (0.1) | 0 | 0 | 0 | 0 | 2 (0.0) |
| LAMA + SABA | 1 (0.2) | 1 (0.1) | 7 (0.6) | 6 (0.7) | 5 (0.8) | 2 (0.3) | 22 (0.4) |
| LAMA + Methylxanthines | 2 (0.3) | 5 (0.5) | 7 (0.6) | 29 (3.2) | 2 (0.3) | 12 (2.0) | 57 (1.1) |
| ICS/LABA + SABA | 4 (0.6) | 18 (1.8) | 6 (0.5) | 16 (1.8) | 21 (3.5) | 4 (0.7) | 69 (1.4) |
| ICS/LABA + Methylxanthines | 5 (0.8) | 10 (1.0) | 9 (0.7) | 81 (9.0) | 3 (0.5) | 22 (3.7) | 130 (2.6) |
| LAMA + ICS/LABA + SABA | 2 (0.3) | 2 (0.2) | 3 (0.2) | 9 (1.0) | 5 (0.8) | 0 | 21 (0.4) |
| LAMA + ICS/LABA + Methylxanthines | 4 (0.6) | 10 (1.0) | 13 (1.0) | 32 (3.5) | 0 | 13 (2.2) | 72 (1.4) |
| LAMA + LABA + ICS | 0 | 0 | 0 | 0 | 0 | 0 | 0 |
| LAMA + LABA + SABA | 0 | 0 | 0 | 0 | 0 | 0 | 0 |
| LAMA + LABA + Methylxanthines | 0 | 0 | 0 | 5 (0.6) | 0 | 1 (0.2) | 6 (0.1) |
| Methylxanthines + SABA | 1 (0.2) | 4 (0.4) | 0 | 9 (1.0) | 1 (0.2) | 11 (1.8) | 26 (0.5) |

^*^ Subjects prescribed one and only mono- or combination of medications on display, with no prohibition of being prescribed mucolytic, traditional Chinese medicine and others at the same time.

COPD, chronic obstructive pulmonary disease; GOLD, Global Initiative for Chronic Obstructive Lung Disease; ICS, inhaled corticosteroid; LABA, long-acting beta_2_-agonist; LAMA, long-acting muscarinic antagonist; SABA, short-acting beta_2_-agonist; SAMA, short-acting muscarinic antagonist.

**Table E7. Distribution of medications prescribed in any form for COPD maintenance by geographical regions**

| Medications used in any form,^*^  n (%) | Northeast  (n = 623) | North  (n = 1005) | East  (n = 1248) | South  Central  (n = 904) | Northwest  (n = 602) | Southwest  (n = 596) | Total  (n = 4978) |
| --- | --- | --- | --- | --- | --- | --- | --- |
| ICS | 4 (0.6) | 16 (1.6) | 1 (0.1) | 8 (0.9) | 2 (0.3) | 3 (0.5) | 34 (0.7) |
| LABA | 3 (0.5) | 15 (1.5) | 74 (5.9) | 43 (4.8) | 6 (1.0) | 36 (6.0) | 177 (3.6) |
| ICS/LABA | 324 (52.0) | 605 (60.2) | 717 (57.5) | 516 (57.1) | 425 (70.6) | 275 (46.1) | 2862 (57.5) |
| SABA | 22 (3.5) | 83 (8.3) | 28 (2.2) | 98 (10.8) | 70 (11.6) | 94 (15.8) | 395 (7.9) |
| SAMA | 5 (0.8) | 49 (4.9) | 17 (1.4) | 38 (4.2) | 1 (0.2) | 1 (0.2) | 111 (2.2) |
| SABA/SAMA | 1 (0.2) | 2 (0.2) | 0 | 6 (0.7) | 4 (0.7) | 1 (0.2) | 14 (0.3) |
| LAMA | 204 (32.7) | 447 (44.5) | 780 (62.5) | 262 (29.0) | 283 (47.0) | 197 (33.1) | 2173 (43.7) |
| Methylxanthines | 120 (19.3) | 71 (7.1) | 46 (3.7) | 274 (30.3) | 27 (4.5) | 167 (28.0) | 705 (14.2) |
| Mucolytics | 81 (13.0) | 204 (20.3) | 108 (8.7) | 224 (24.8) | 17 (2.8) | 151 (25.3) | 785 (15.8) |
| TCM | 17 (2.7) | 159 (15.8) | 127 (10.2) | 72 (8.0) | 103 (17.1) | 100 (16.8) | 578 (11.6) |
| Others | 37 (5.9) | 189 (18.8) | 207 (16.6) | 310 (34.3) | 52 (8.6) | 156 (26.2) | 951 (19.1) |
| Neither ICS nor long-acting bronchodilator^†^ | 155 (24.9) | 130 (12.9) | 39 (3.1) | 196 (21.7) | 39 (6.5) | 122 (20.5) | 681 (13.7) |

^*^ Medications classified by drug class; ^†^ this class denotes prescriptions without ICS, LABA, ICS/LABA or LAMA.

COPD, chronic obstructive pulmonary disease; GOLD, Global Initiative for Chronic Obstructive Lung Disease; ICS, inhaled corticosteroid; LABA, long-acting beta_2_-agonist; LAMA, long-acting muscarinic antagonist; SABA, short-acting beta_2_-agonist; SAMA, short-acting muscarinic antagonist; TCM, traditional Chinese medicine.
